# The Use of Mobile Applications for the Diagnosis and Treatment of Tumors in Orthopaedic Oncology – a Systematic Review

**DOI:** 10.1007/s10916-021-01774-z

**Published:** 2021-10-09

**Authors:** J. Berger-Groch, M. Keitsch, A. Reiter, S. Weiss, KH. Frosch, M. Priemel

**Affiliations:** 1 Department of Trauma and Orthopaedic Surgery, University Medical Center Hamburg-Eppendorf, Martinistr. 52, 20246 Hamburg, Germany; 2 Department of Trauma Surgery, Orthopaedics and Sports Traumatology, BG Hospital Hamburg, Bergedorfer Str. 10, 21033 Hamburg, Germany

**Keywords:** mHealth, Smartphones, Sarcoma, App, Bone tumor, Mobile application

## Abstract

The use of smartphone apps is an essential part of everyday life. Mobile applications offer enormous opportunities for dealing with challenges in public health, and their number increases every day. This paper aims to review the existing literature on mobile applications in orthopaedic oncology and to summarize the current mobile applications for musculoskeletal tumors. A systematic literature review was conducted regarding articles on mobile applications in orthopaedic and trauma surgery. The focus was on identifying mobile applications that can be used in the treatment of patients with musculoskeletal tumors. Two reviewers independently assessed study eligibility, extracted data, and appraised methodological quality. In addition, the Apple App Store and Google Play Store were searched for suitable mobile applications. Ninety-one articles describing a mobile application in orthopaedic and trauma surgery were identified. Three articles focused on a mobile application for musculoskeletal tumors. Additionally, seven mobile applications were available in the App/Play Stores dealing with bone or soft tissue tumors in orthopaedic oncology without corresponding scientific articles. Increasing numbers of mobile applications are being developed in orthopaedic and trauma surgery. Currently, only three scientific articles on mobile applications in orthopaedic oncology are present, yet several more applications are available without scientific medical evaluation. Since mobile applications can facilitate the everyday life of orthopaedic and trauma surgeons, it is worthwhile to be aware of new developments in this field. A regular scientific evaluation of the subject is important in order to classify the significance of these applications.

**Supplementary information:**

The online version contains supplementary material available at 10.1007/s10916-021-01774-z.

## Introduction

Digital transformation is a dynamic process that includes the whole society. Almost all hospitals in Europe are converting their documentation from paper files to digital files as part of the digitization process [1]. While it is hard to imagine everyday life without smartphones, the use of mobile phone applications in everyday medical practice is still scarce [2].

In recent years, more and more medical mobile applications were developed [3]. The largest share of mobile applications currently available are so-called “health apps” or “lifestyle apps” such as mobile applications to help users lose weight or monitor the daily exercise [4]. Most of these applications are targeted to be used by non-medical personnel in their private life. Nevertheless, some mobile applications are already available for and by physicians.

The development of medical apps does not necessarily involve physicians at all and medical mobile applications can be published in the app stores without being checked for medical accuracy, relevance or correctness. So the evaluation of reliability and accuracy of the content of healthcare-related mobile applications is important, as the range of available mobile applications is so dynamic that their quantity and quality varies every day [5].

The field of medical smartphone applications is summarized under the keyword *mHealth*, which describes the use of smartphones and connected devices in a health care context [6]. While the development of new mobile applications for the specialty of orthopaedic and trauma surgery is considered a future-oriented matter with a lot of potentials, it still seems underrepresented in this field [4].

The treatment of musculoskeletal tumors remains a challenge for orthopaedic and trauma surgeons and should be performed at specialized centers. Nevertheless, the initial diagnosis of musculoskeletal tumors is an everyday challenge for non-specialized physicians in facilities of any level of care. Especially for rare diseases, the support of physicians by mHealth applications is beneficial [7]. The aim of this review is to give orthopaedic or trauma surgeons an overview of currently available literature about mobile medical applications with the focus on the diagnosis and treatment of musculoskeletal tumors and to present available mobile applications.

## Materials and methods

**Fig. 1:**
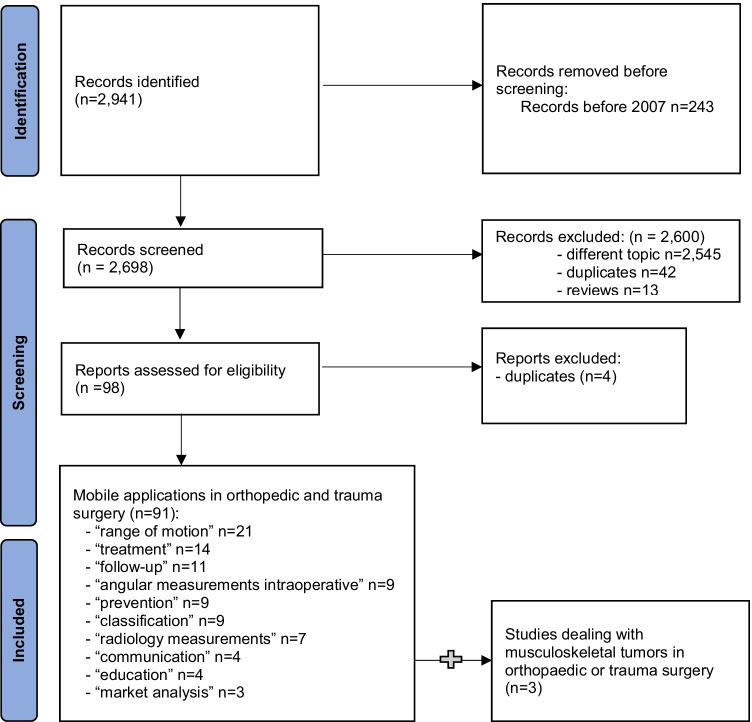
PRISMA flow diagram

To evaluate the use of mobile applications for the treatment of bone tumors in orthopaedic and trauma surgery, we performed a systematic review of the available literature. A systematic literature review was conducted using the Pubmed / Medline OVID database and Cochrane library database on February, 27^th^ 2021. The following search string was used for this purpose: (mobile application and tumor) OR (mobile application and bone) OR (mobile application and trauma) OR (mobile application and fracture) OR (smartphone and tumor) OR (smartphone and bone) OR (smartphone and trauma) OR (smartphone and fracture) (see Fig. 1 PRISMA Flow Chart). First, the titles and abstracts of all records were reviewed.

The PRISMA Checklist 2009 was followed for assessment of systematic reviews. All original research studies focusing on a mobile application in the field of orthopaedic and trauma surgery and the diagnosis or treatment of bone tumors were included. In 2007, the first iPhone® was presented. Due to the technological development, studies published before January 2007 were excluded. Only full-text articles written in English or German were included. Editorials, pre-prints, and conference abstracts without full-text articles were not eligible for inclusion. There was no limitation regarding patient age, gender, or the number of included patients.

Articles focusing on other specialties (e.g. dermatology) were excluded as well as studies focusing and describing non-mobile applications. Articles focusing on a mobile application in orthopaedic and trauma surgery but not on musculoskeletal tumors were excluded in the last step.

All articles were screened for eligibility by two independent authors (MK, JBG). Inclusion and exclusion decisions were based on a group consensus agreement.

Each article was evaluated for methodological quality by two authors separately (MK, JBG). The consensus was achieved when discrepancies between scores were found. The Methodological Index for Non-randomized Studies (MINORS) [8] was used for all articles and the Jadad scale [9] for randomized studies only. Higher scores indicate higher methodological quality.

The MINORS score was developed for nonrandomized studies in the field of surgery. The score consists out of 8 (single arm studies) or rather 12 questions (comparative study) (see Supplement 1). The maximum score for comparative studies is 24 and for single-arm studies 16. Each question can be rated with 0 (not reported), 1 (reported but inadequate), or 2 (reported and adequate) points. A score ≥ 20 for comparative studies and ≥ 12 for single-arm studies indicate a low risk of bias [10].

The Jadad scale favors studies that are well-randomized and blinded, assessing the appropriateness of randomization and blinding [9]. The modified Jadad scale consisting of 6-items was used [11]. Scale scores range from 0 to 8 points, with higher scores indicating better quality. 0–3 points signified low-quality studies, while 4–8 points signified high-quality studies [11] (see Supplement 2).

Technical notes were excluded when analyzing methodological quality.

Search results were sorted by survey studies (user/market analysis), technical notes (app presentation), comparative studies (comparison of app vs. other app or another tool), and randomized controlled trials (RCTs). Additional relevant reviews were screened for articles fitting the inclusion criteria.

In addition to the literature review, an analysis of currently available mobile applications designed for orthopaedic and trauma surgeons in the *Apple App Store* and *Google Play Store* was performed on April 3^rd^, 2021. The following search terms in English and German were used: “tumor/tumour”, "bone tumor/tumour”, “bonetumor/tumour”, “orthopaedics/orthopaedics”, “trauma surgery”, “Knochentumor”, “Orthopädie”, “Unfallchirurgie”. Relevant mobile applications were classified according to their topic and target group. Furthermore, it was recorded how many times the mobile application was downloaded, its rating and price, and whether there was any indication of professional medical co-development.

## Results

### Literature research

The search string resulted in 2,941 identified records. 2,545 articles were excluded as they did not deal with a mobile application or focused on a mobile application in a medical field other than orthopaedic or trauma surgery. Ninety-eight original studies focused on mobile applications in orthopaedic and trauma surgery in general. Four duplicate studies were removed. The ninety-four remaining articles were divided as follows: forty-five comparative studies, thirty-two technical notes, twelve randomized controlled trials, and five survey studies. Three articles described a mobile application regarding musculoskeletal tumors.

The three studies that address smartphone applications in tumor orthopaedics are two technical notes describing the possibility of using augmented reality via the phone’s camera to excise a tumor [7, 12] and another technical note about a mobile application developed as a post-surgery tracking-system for patients with musculoskeletal tumors [13]. As the three articles are technical notes no analysing of methological quality according to MINOR criteria was performed.

To estimate the quality of existing articles on mobile applications in orthopedics and trauma surgery (n = 91) in general a methodological quality assessment was performed. Technical notes (n = 29) were excluded. As one of the twelve randomized trials described a planned study, this paper was excluded for quality assessment [14]. From the remaining sixty-two articles, the overall mean MINORS score was 14.8 ± 4.4 (range 4.0–24.0). In total, 16 studies (25%) had a MINORS score of higher than 16.0, which has been regarded as the cut-off for a high-quality study [15]. The Jadad scale for the randomized trials was 5.3 ± 0.9 (range 4.0–7.0).

Among the ninety-one original papers, twenty-one mobile applications dealt with the determination of angles in clinical examinations (range of motion (ROM) or misalignments), fourteen mobile applications on treatment pathways in orthopaedic or trauma surgery, eleven mobile applications focused on follow-up examination, nine on intraoperative angular measurements, nine on prevention, nine on classification, seven on radiology measurements, four on communication, four on education and three on market analysis (survey).(see Supplement 3).

Eleven randomized controlled trials were identified (see Table 1). They were focused on the investigation of methods improving the follow-up treatment of patients with or without mobile application support. None of these articles dealt with oncologic, orthopaedic surgery. Ten trials were related to applications that were designed for patients. Only one mobile application was not designed for patients and served to support medical staff. High-quality articles regarding the use of mobile applications by physicians in their daily routine were completely absent.

**Table 1:** Overview of the 11 randomized trials identified on the topic of mobile applications in orthopaedic and trauma surgery

first author	name of publication	category	year of publication	user
Svingen	A smartphone application to facilitate adherence to home-based exercise after flexor tendon repair: A randomised controlled trial	follow-up	2021	patient
Higgins	Conventional Follow-up Versus Mobile Application Home Monitoring for Postoperative Anterior Cruciate Ligament Reconstruction Patients: A Randomized Controlled Trial	follow-up	2020	patient
Li	Effects of a home-based occupational therapy telerehabilitation via smartphone for outpatients after hip fracture surgery: A feasibility randomised controlled study	follow-up	2020	patient
Blanquero	Feedback-guided exercises performed on a tablet touchscreen improve return to work, function, strength and healthcare usage more than an exercise program prescribed on paper for people with wrist, hand or finger injuries: a randomised trial	treatment	2020	patient
Ryan	Efficacy of Osteoporosis Prevention Smartphone App	prevention	2020	patient
Chhabra	Smartphone app in self-management of chronic low back pain: a randomized controlled trial	treatment	2018	patient
Hardt	Improved early outcome after TKA through an app-based active muscle training programme-a randomized-controlled trial	follow-up	2018	patient
Morkeberg Nilsson	Cost-Effectiveness of Mobile App-Guided Training in Extended Focused Assessment with Sonography for Trauma (eFAST): A Randomized Trial	education	2017	medical stuff
Van Reijen	The "Strengthen your ankle" program to prevent recurrent injuries: A randomized controlled trial aimed at long-term effectiveness	prevention	2017	patient
Park	Application and Effect of Mobiletype-Bone Health Intervention in Korean Young Adult Women with Low Bone Mass: a Randomized Control Trial	prevention	2017	patient
Irvine	Mobile-Web app to self-manage low back pain: randomized controlled trial	treatment	2015	patient

### App store research

The analysis of the Google Play Store revealed five mobile applications dealing with musculoskeletal tumors. (see Table 2) There were all free of charge. The mobile application with the most significant number of downloads was the “Bone and soft tissue tumors case studies” (BoSTT) app. This is a mobile source of musculoskeletal tumor cases for medical education. It is designed for all healthcare professionals working in the field of musculoskeletal healthcare. The cases are provided by experts at the Royal National Orthopedic Hospital (RNOH) NHS Trust, Stanmore, UK.

**Table 2:** Overview of the five mobile applications identified in the Google Play Store on the topic of musculoskeletal tumors in orthopaedic and trauma surgery

Name	Topic	Target group	Down-loads (04/2021)	Rating	Price	Professional co-development	published	updated
BoSTT	source of musculoskeletal tumour cases	healthcare professional	over 5000	4,2/5	0	yes	2015	2020
MSK Oncology Educational Atlas	interactive learning atlas for orthopaedic oncology	healthcare professional	over 500	none	0	yes	2017	2017
Personalized Sarcoma Care	information tool on the treatment options of sarcoma and survival rates	patient and healthcare professional	over 500	4,5/5	0	yes	2017	2020
Lipoma Disease	information on lipoma	patients	over 1000	none	0	unclear	2017	2017
Recognize lipoma disease	information on lipoma	patients	over 100	none	0	unclear	2017	2019

A further learning tool for healthcare professionals is the “MSK oncology educational atlas” app. The mobile application is an interactive educational tool focusing on the most common topics related to orthopaedic oncology. It highlights important aspects of benign and malignant bone and soft tissue tumors in 45 self-paced content modules and finishes with self-assessment multiple choice questions. It was developed by the Department of Orthopedic Surgery at University of Michigan, USA.

Third, a mobile application called “Personalized sarcoma care” was identified, which enables physicians and patients to determine the survival of sarcoma patients. It is a prognostic tool specifically designed to support shared decision making for patients with primary high-grade soft tissue sarcoma in their limbs. Using patient- and tumour-related characteristics, the mobile application provides an estimate of the oncological outcome in terms of overall survival or incidence of local recurrence. The development of the mobile application was done by Leiden University Medical Center (LUMC), Netherlands. This mobile application is also available in the Apple App Store but was not shown under the defined search string for this study.

Finally, two mobile applications (“Lipoma Disease”; “Recognize lipoma disease”) were found that provide information about lipomas. These mobile applications are designed for use by patients and do not provide any relevant insight from a scientific point of view. The involvement of a physician in the development of both mobile applications cannot be deducted from the published information.

The analysis of the Apple App Store revealed four mobile applications dealing with musculoskeletal tumors. (see Table 3) They were all free of charge. BoSTT and MSK Oncology Educational Atlas were the same as in Google Play Store (see above).

**Table 3:** Overview of the four mobile applications identified in the Apple App Store on the topic of musculoskeletal tumors in orthopaedic and trauma surgery

Name	Topic	Target group	Down-loads (04/21)	Rating	Price	Professional co-development	published	updated
BoSTT	source of musculoskeletal tumour cases	healthcare professional	n.a	n.a	0	yes	2015	2020
MSK Oncology Educational Atlas	interactive learning atlas for orthopaedic oncology	healthcare professional	n.a	n.a	0	yes	2017	2018
OOLH sarcoma care	educating users regarding orthopaedic oncology and provides curated information regarding the services provided	Patient and healthcare professionals	n.a	n.a	0	yes	2018	2018
MeVis Recist	evaluate therapy response in the treatment of solid tumors	Healthcare professional	n.a	5/5	0	yes		2020

The OOLH sarcoma care mobile application aims to introduce programs, services, and achievements to citizens of Thailand with the purpose of educating users regarding orthopaedic oncology and provide information. The OOLH mobile application has a forum for health professionals to discuss cases.

The *MeVis Recist* app Response evaluation criteria in solid tumors (RECIST) is a set of published rules that define when tumors in cancer patients improve ("respond"), stay the same ("stabilize"), or worsen ("progress") during treatment. The mobile application helps to define the RECIST calculation. This mobile application is also available in the Google Play Store, but was not shown with the entered search string.

## Discussion

This systematic review identified ninety-one articles dealing with mobile applications in orthopaedic or trauma surgery. Three articles focussed on a smartphone application for musculoskeletal tumors in trauma or orthopaedic surgery. Two articles dealt with augmented reality to support tumor resection [7, 12]. One was developed to improve the follow-up of patients with musculoskeltal tumors [13]. Seven mobile applications were available in the Apple App Store and Google Play Store dealing with bone or soft tissue tumors in trauma or orthopaedic surgery. Nearly all available mobile applications are concerned with conveying learning content or imparting knowledge. So far, no application is available that actively supports the physician in finding a diagnosis or suggests therapeutic options for a specific case. Only one mobile application to evaluate the prognosis for sarcoma patients is available. The potential for new apps to be developed is great. According to Dittrich et al., 62.5% of users in orthopaedic and trauma surgery were dissatisfied with the current range of medical mobile applications in general [16].

Consistent with our results, Wong et al. described that patient education and exercise programs made up the largest share of available applications. Applications for medical professionals are mainly used for education and as measuring tools [4]. Measurement tools are an important instrument for orthopaedic and trauma surgeons to enhance clinical examinations. In particular, this is also reflected in the number of identified articles in the present review. 41% (31/76) of all reviewed articles dealt with examination of the range of motion, intraoperative angle determination, or the determination of angles on imaging techniques. Some innovative concepts are presented, such as the description of a smartphone applications to adjust cup inclination angles during total hip arthroplasty [17] or a tool to improve the pivot shift test with a smartphone accelerometer [18].

This literature review, especially based on the many existing technical notes, showed that there are countless possibilities and technical opportunities to support physicians in their everyday work via smartphone applications. Nevertheless, the final step that takes an individual mobile application from prototype to a product for the masses seems to be difficult. The creation of a mobile application depends on appropriate funding and a large team to comply with requirements of medical devices, data security, and providing ongoing technical support. Wong et al. identified 76 individual mobile applications for physicians and patients in orthopaedic sports medicine. They highlighted that only 39% had named medical professional involvement in their development or content [4]. In order to obtain a high-quality product, the involvement of physicians is indispensable in this process [16].

However, projects that are supported by the appropriate organizations can be very valuable for physicians, whether in training or in everyday work. This can be seen, for example, in the BoSTT mobile application, where with the background support of a large hospital, orthopaedic and trauma surgeons share their knowledge with users. New cases with radiological and pathological images of musculoskeletal tumors are continuously posted every month. Users have the chance to think about the respective clinical image and receive the most important learning points briefly and concisely. The increasing acceptance of smartphones as an aid in the professional environment will hopefully raise the interest to evaluate scientifically fundable projects in larger settings and examine their value and impact.

One reason why physicians do not use cell phones in everyday life as they do in their private lives could be seen in data security issues, but also in the question of reputation. In many clinics, for example, it is not permitted to take photos or videos with a private cell phone due to non-compliance with data security laws. Blocker et al. highlighted the concern that patients and colleagues might quickly cast a critical eye on someone using a smartphone at work [19]. To eliminate such fears, clear data security concepts are necessary. A web-based survey among 206 orthopaedic and trauma surgeons in Germany identified data misuse as the greatest perceived risk regarding the use of mHealth mobile applications [16]. The use of devices intended only for work could present a solution to this issue.

An example of such a device is the so called NIDApad® (medDV, Fernwald, Germany) introduced in emergency medicine [20]. If necessary, these tablets can take photos of the accident scene in order to pass on relevant information to the other colleagues without being stored private cell phones. Another concept is the “PhotoExam App” used at all Mayo Clinics. Videos are stored on the local device only temporarily until they are uploaded or the user closes the mobile application, then they are deleted permanently from the user´s device [21].

Another reason for the still restrained use of smartphones in the daily work of an orthopaedic or trauma surgeon is the poor visibility of relevant mobile applications in App Stores. Several mobile applications described in the included articles were not available under the specified search criteria in App Stores [7, 14, 22]. When publishing a new application, this should be taken into account.

## Conclusion

An increasing number of mobile applications are being developed in orthopaedic and trauma surgery in general. The potential options to support the physician in the diagnosis, treatment or therapy of musculoskeletal tumors by means of a mobile application are numerous. In the moment only three scientific articles are dealing with mobile applications helping in the field of orthopaedic oncology, but several more mobile applications are already available. As these tools can serve as an important aid, surgeons need to be aware of the opportunities offered by these tools. However, not only the surgeon but also the hospital infrastructure decision-makers (IT, data protection officer, purchasing, etc.) must be involved in the process of implementing apps in everyday hospital life in order to make secure data use possible.

## Supplementary information

Below is the link to the electronic supplementary material.

Supplementary file1 (DOCX 28 KB)

## Data Availability

Data are available on request from the corresponding author.
