# Supplementary material for: The Use of Mobile Applications for the Diagnosis and Treatment of Tumors in Orthopaedic Oncology – a Systematic Review
Source: J Med Syst. 2021 Oct 9;45(11):99. doi: 10.1007/s10916-021-01774-z (PMC8502123; doi:10.1007/s10916-021-01774-z)
Supplement: Supplementary file 1 — Supplementary file1 (DOCX 28 KB) [file 10916_2021_1774_MOESM1_ESM.docx]

Supplements:

| **MINORS** |  |
| --- | --- |
| 1 | **A clearly stated aim:** the question addressed should be precise and relevant in the light of available literature |
| 2 | **Inclusion of consecutive patients** : all patients potentially fit for inclusion (satisfying the criteria for inclusion) have been included in the study during the study period (no exclusion or details about the reasons for exclusion) |
| 3 | **Prospective collection of data**: data were collected according to a protocol established before the beginning of the study |
| 4 | **Endpoints appropriate to the aim of the study**: unambiguous explanation of the criteria used to evaluate the main outcome which should be in accordance with the question addressed by the study. Also, the endpoints should be assessed on an intention-to-treat basis |
| 5 | **Unbiased assessment of the study endpoint**: blind evaluation of objective endpoints and double-blind evaluation of subjective endpoints. Otherwise the reasons for not blinding should be stated |
| 6 | **Follow-up period appropriate to the aim of the study**: the follow-up should be sufficiently long to allow the assessment of the main endpoint and possible adverse events |
| 7 | **Loss to follow up less than 5%**: all patients should be included in the follow up. Otherwise, the proportion lost to follow up should not exceed the proportion experiencing the major endpoint |
| 8 | **Prospective calculation of the study size**: information of the size of detectable difference of interest with a calculation of 95% confidence interval, according to the expected incidence of the outcome event, and information about the level for statistical significance and estimates of power when comparing the outcomes |
| *Additional criteria in the case of comparative study* | |
| 9 | **An adequate control group**: having a gold standard diagnostic test or therapeutic intervention recognized as the optimal intervention according to the available published data |
| 10 | **Contemporary groups**: control and studied group should be managed during the same time period (no historical comparison) |
| 11 | **Baseline equivalence of groups**: the groups should be similar regarding the criteria other than the studied endpoints. Absence of confounding factors that could bias the interpretation of the results |
| 12 | **Adequate statistical analyses**: whether the statistics were in accordance with the type of study with calculation of confidence intervals or relative risk |
| 0 (not reported), 1 (reported but inadequate) or 2 (reported and adequate).  The global ideal score being 16 for non-comparative studies and 24 for comparative studies. | |

Supplement 1: Overview of the 12 questions needed to create the MINOR criteria as published by: Slim K, Nini E, Forestier D, Kwiatkowski F, Panis Y, Chipponi J. Methodological index for non-randomized studies (minors): development and validation of a new instrument. *ANZ J Surg*. 2003;73(9):712-716. doi:10.1046/j.1445-2197.2003.02748.x

| **6-item Jadad scale** |  |  |
| --- | --- | --- |
| 1a. Was the study described as randomized? | Yes | +1 |
|  | No | 0 |
| 1b. Was the method of randomization appropriate? | Yes | +1 |
|  | No | -1 |
|  | Not described | 0 |
| 2a. Was the study described as double-blinded? | Yes | +1 |
|  | No | 0 |
| 2b. Was the method of double-blinding appropriate? | Yes | +1 |
|  | No | -1 |
|  | Not described | 0 |
| 3. Was there a description of withdrawals and dropouts? | Yes | +1 |
|  | No | 0 |
| 4. Was there a clear description of the inclusion/exclusion criteria? | Yes | +1 |
|  | No | 0 |
| 5. Was the method used to assess adverse effects described? | Yes | +1 |
|  | No | 0 |
| 6. Was the method of statistical analysis described? | Yes | +1 |
|  | No | 0 |

Supplement 2: Overview of the 6 questions needed to create the modified Jadad scale as published by: Oremus M, Wolfson C, Perrault A, Demers L, Momoli F, Moride Y. Interrater reliability of the modified Jadad quality scale for systematic reviews of Alzheimer’s disease drug trials. *Dement Geriatr Cogn Disord*. 12(3):232-236. doi:10.1159/000051263

| **title** | **year** | **content** |
| --- | --- | --- |
| Impact of a Streamlined Trauma-Focused Smartphone Application on Protocol Compliance and Delivery of Care. | 2020 | app for residence to improve treatment of trauma patients |
| Effects of a home-based occupational therapy telerehabilitation via smartphone for outpatients after hip fracture surgery: A feasibility randomised controlled study. | 2020 | practice at home after total hip arthoplasty |
| Development and Validation of a Mobile Application for Measuring Femoral Anteversion in Patients With Cerebral Palsy. | 2020 | app to measure the angles of the femur |
| Efficacy of Osteoporosis Prevention Smartphone App. | 2020 | app for prevention of osteoporosis |
| A New Fracture Liaison Service Using the Mobile Application and IoT Sensor | 2019 | secondary fracture prevention program for osteoporotic fracture patients |
| Wound Care Follow-Up From the Emergency Department Using a Mobile Application: A Pilot Study. | 2019 | app for smartphone application-based follow-up after wound care in the ED |
| The reliability of use of WhatsApp in type 1 and type 2 pediatric supracondylar fractures. | 2019 | evaluate the reliability of the assessment of radiological X-ray images of traumatic injuries in the elbows of children using WhatsApp application, compared to true-size images on a Picture Archiving and Communication System (PACS) screen |
| Smart Trauma: Improving the Delivery of Evidence-Based Trauma Care. | 2019 | app that presents the latest guidelines for trauma surgeons |
| The validity and reliability of DrGoniometer, a smartphone application, for measuring forearm supination. | 2019 | reliability and validity of DrGoniometer (CDM S.r.L, Cagliari, Italy) for measuring forearm supination in healthy populations and those with forearm fractures |
| Reliability of Smartphone-Based Instant Messaging Application for Diagnosis, Classification, and Decision-making in Pediatric Orthopedic Trauma. | 2019 | reliability of smartphone-based instant messaging applications for the evaluation of various pediatric limb traumas, as compared with the standard method of viewing images of a workstation-based picture archiving and communication system (PACS) |
| Validity and reliability of smartphone orientation measurement to quantify dynamic balance function. | 2018 | development a novel smartphone-based neuromotor assessment protocol for screening of dynamic balance decrements stemming from head trauma |
| The smartphone inclinometer: A new tool to determine elbow range of motion? | 2018 | 3 methods to measure flexibility of the elbow |
| Application and Effect of Mobiletype-Bone Health Intervention in Korean Young Adult Women with Low Bone Mass: a Randomized Control Trial | 2017 | app to improve bone health |
| Heightened clinical utility of smartphone versus body-worn inertial system for shoulder function B-B score | 2017 | comparison of two methods to measure the flexibility of the shoulder |
| Comparison of PACS and Bone Ninja mobile application for assessment of lower extremity limb length discrepancy and alignment. | 2016 | assessment of the accuracy of limb deformity measurements on the Bone Ninja app compared to PACS and testing the intra- and inter-observer variability among different orthopaedic practitioners |
| Reliability and Effectiveness of Smartphone Technology for the Diagnosis and Treatment Planning of Pediatric Elbow Trauma | 2016 | examination whether remote diagnosis of pediatric elbow fractures using smartphone technology is reliable compared to PACS |
| Digital health technology and trauma: development of an app to standardize care | 2015 | describes the process of developing an app, which includes standardized trauma algorithms; the objective of the app was to allow easy, real-time access to trauma algorithms, and therefore reduce omissions/errors |
| The Mersey Burns App: evolving a model of validation. | 2015 | presents two studies assessing the speed and accuracy of calculations using Mersey Burns (App) - which aids in the assessment of total burn surface area (TBSA) and calculation of fluid resuscitation protocols in burns - in comparison with a Lund and Browder chart (paper) when a burn is assessed by medical students and clinicians |
| Smartphone surgery: how technology can transform practice. | 2014 | description of initial teletrauma experience and the effect of smartphone implementation in patient care and medical education at the University of Arizona Medical Center in Tucson |
| Feedback-guided exercises performed on a tablet touchscreen improve return to work, function, strength and healthcare usage more than an exercise program prescribed on paper for people with wrist, hand or finger injuries: a randomised trial | 2020 | comparison of feedback-guided exercises performed on a tablet touchscreen compared to a home exercise program prescribed on paper regarding return to work, healthcare usage and clinical recovery |
| Interobserver variability of radiographic assessment using a mobile messaging application as a teleconsultation tool | 2017 | comparison of interobserver reliability, decision-making, and confidence in decision-making in the treatment of distal radius fractures depending on wheather radiographs are viewed on a messenger application on a mobile phone or with a standard DICOM viewer. |
| Evaluation of a Mobile Application for Multiplier Method Growth and Epiphysiodesis Timing Predictions | 2017 | comparison of the traditional multiplier method (MM) to predict limb-length discrepancy and timing of epiphysiodesis with am mobile application that was developed in an attempt to simplify and streamline the calculations |
| Reliability of a Smartphone Goniometric Application in the Measurement of Hip Range of Motion Among Experienced and Novice Clinicians | 2021 | app to measure the flexibility of the hip |
| Technical Note on Using Intraoperative Smartphone Applications to Adjust Cup Inclination Angle during Total Hip Arthroplasty (THA) | 2020 | app to measure the Cup Inclination Angle during Total Hip Arthroplasty |
| Smartphone and App Usage in Orthopedics and Trauma Surgery: Survey Study of Physicians Regarding Acceptance, Risks, and Future Prospects in Germany | 2020 | market analysis regarding usage of mobile applications in trauma care and orthopedics |
| A smartphone application to facilitate adherence to home-based exercise after flexor tendon repair: A randomised controlled trial | 2021 | app for follow -up after flexor tendon repair compared to standard rehabilitation |
| Mobile App for Monitoring 3-Month Postoperative Functional Outcome After Hip Fracture: Usability Study | 2020 | app for monitoring functional outcome after hip fracture |
| BAck iN the Game (BANG) - a smartphone application to help athletes return to sport following anterior cruciate ligament reconstruction: protocol for a multi-centre, randomised controlled trial | 2020 | randomized trial that tests whether a custom smartphone application delivering cognitive-behavioural therapy is effective for improving the number of people who return to their preinjury sport and level following ACL reconstruction |
| Child concussion recognition and recovery: a community delivered, evidenced-based solution | 2020 | app for management of concussion in children aged 5-18 years. The application consists of (I) a sideline concussion check and (II) symptom monitoring and symptom-targeted psychoeducation to assist the parent manage their child's safe return to school, exercise and sport |
| Enhancing Trauma Patient Experience Through Education and Engagement: Development of a Mobile Application | 2020 | patient education app with information regarding injury, treatment, and recovery for orthopaedic and other injuries |
| Conventional Follow-up Versus Mobile Application Home Monitoring for Postoperative Anterior Cruciate Ligament Reconstruction Patients: A Randomized Controlled Trial | 2020 | randomized trial to determine whether a mobile app can reduce the need for in-person visits and examine the resulting societal cost differences between mobile and conventional follow-up for postoperative anterior cruciate ligament (ACL) reconstruction patients. |
| A Possible Mobile Health Solution in Orthopedics and Trauma Surgery: Development Protocol and User Evaluation of the Ankle Joint App | 2020 | protocol of the development process an user evaluation of the Ankle Joint App, an app that provides medical literature, training videos and a log function regarding ankle sprains |
| Quantitative assessment of the pivot shift test with smartphone accelerometer | 2020 | app to assess the Pivot Shift phenomenon |
| WhatsApp Mobile Health Platform to Support Fracture Management by Non-Specialists in South Africa | 2020 | assessment of the communication between Non-orthopaedic doctors and an orthopedic referral group via whatsapp |
| Development of a System for Real-Time Monitoring of Pressure, Temperature, and Humidity in Casts | 2019 | app to document pressure, temperature and humidity in casts |
| myHip&Knee: Improving Patient Engagement and Self-Management Through Mobile Technology | 2019 | app to improve patient self-management after knee or hip arthroplasty to reduce follow-up calls to surgeons' offices, ultimately reducing demand on healthcare resources |
| Reliability of a Smartphone Compared With an Inertial Sensor to Measure Shoulder Mobility: Cross-Sectional Study | 2019 | meassurement of shoulder mobility with an app compared to measurement with an inertial sensor |
| Evaluation of the smartphone for measurement of femoral rotational deformity | 2019 | measure the femoral rotational deformity with a smartphone |
| Mobile VR-Application for Neck Exercises | 2019 | use of a VR-based App for treatment, rehabilitation and prevention of neck injuries |
| Reliability and concurrent validity of a mobile application to measure thoracolumbar range of motion in low back pain patients | 2020 | verify the validity of thoracolumbar ROM using a mobile application and a digital inclinometer |
| Smartphone applications for the evaluation of pathologic shoulder range of motion and shoulder scores-a comparative study | 2018 | the aim of this study was to establish the reliability and validity of different smartphone applications in assessing pathologic shoulder ROM and to determine whether differences in recorded ROM measurements affect calculated shoulder scores |
| Design and validation of a smart wearable device to prevent recurrent ankle sprain | 2018 | smart wearable device to prevent recurrent ankle sprain |
| Help at hand: Women's experiences of using a mobile health application upon diagnosis of asymptomatic osteoporosis | 2018 | testing a mobile health application developed for women newly diagnosed with asymptomatic osteoporosis regarding whether the app can help them to be prepared for treatment decision-making and support them in osteoporosis self-management. |
| Planning a total knee arthroplasty through an application for mobile devices: case report | 2018 | planning Total Knee Arthroplastie with the help of an app |
| Smartphone app in self-management of chronic low back pain: a randomized controlled trial | 2018 | app for self-management for patients with chronic back pain |
| Using a Mobile Application to Assess Knee Valgus in Healthy and Post-Anterior Cruciate Ligament Reconstruction Participants | 2019 | mobile Application to Assess Knee Valgus in Healthy and Post-Anterior Cruciate Ligament Reconstruction Participants |
| A New, Easy, Fast, and Reliable Method to Correctly Classify Acetabular Fractures According to the Letournel System | 2018 | app to Classify Acetabular Fractures According to the Letournel System |
| Evaluation of benefits and accuracy of a mobile application in planning total knee arthroplasties | 2018 | evaluation of benefits and accuracy of a mobile application in planning total knee arthroplasties |
| The CJOrtho app: A mobile clinical and educational tool for orthopedics | 2018 | app to provie fast access to 65 classification systems in orthopedics or trauma surgery, 20 clinical outcome scores and a digital goniometer. |
| Improved early outcome after TKA through an app-based active muscle training programme-a randomized-controlled trial | 2018 | app-based muscle training to improve the outcome after total knee arthroplasty |
| Is the pelvis stable during supine total hip arthroplasty? | 2017 | app to measure pelvic position during total hip arthroplasty |
| Usefulness and Radiological Evaluation of Accuracy of the Innovative "Smart" Hand Technique for Pedicle Screw Placement: An Anatomical Study | 2018 | app to naviate Pedicle Screw Placement |
| The relationship between the use of running applications and running-related injuries | 2018 | evaluation of the relationship between the use of running applications and running related injuries |
| An eHealth Application of Self-Reported Sports-Related Injuries and Illnesses in Paralympic Sport: Pilot Feasibility and Usability Study | 2017 | app to list self-reported injuries and illnesses in paralympic sports |
| Apps in Trauma and Emergency Surgery | 2017 | a digital survey was designed to explore the usage of mobile smartphones and the associated apps among surgeons in Trauma and Emergency departments |
| Development of an mHealth trauma registry in the Middle East using an implementation science framework | 2017 | development of a tablet-based trauma registry in the Middle East |
| Fall Detection in Individuals With Lower Limb Amputations Using Mobile Phones: Machine Learning Enhances Robustness for Real-World Applications | 2017 | mobile phone-based fall detection model was developed using data from able-bodied individuals to detect falls in individuals with a lower limb amputation, while they freely carry the mobile phone in different locations and during free-living |
| Cost-Effectiveness of Mobile App-Guided Training in Extended Focused Assessment with Sonography for Trauma (eFAST): A Randomized Trial | 2017 | evaluation of the cost-effectiveness of mobile app-guided versus textbook-guided ultrasound training |
| REPRODUCIBILITY OF SCHATZKER CLASSIFICATION THROUGH SMARTPHONE APPLICATIONS | 2016 | evaluation of the intra-observer reproducibility of Schatzker classification for tibial plateau fractures through smartphone applications |
| A new iPhone application for measuring active craniocervical range of motion in patients with non-specific neck pain: a reliability and validity study | 2018 | iphone App to determine the craniocervical range of motion in patients with unspecific neck pain |
| Reducing concussion symptoms among teenage youth: Evaluation of a mobile health app | 2017 | evaluation whether a mobile health application that employs elements of social game design could compliment medical care for unresolved concussion symptoms compared to standard of care for concussion alone. |
| The "Aachen fall prevention App" - a Smartphone application app for the self-assessment of elderly patients at risk for ground level falls | 2017 | app for elderly patientsto to self-assess and monitor their individual fall risk |
| The Use of WhatsApp Smartphone Messaging Improves Communication Efficiency within an Orthopaedic Surgery Team | 2017 | report on the use of WhatsApp to invetsigate Communication Efficiency within an Orthopaedic Surgery Team |
| Cost-Effective Mobile-Based Healthcare System for Managing Total Joint Arthroplasty Follow-Up | 2017 | development of a mobile-based healthcare system that provides cost-effective follow-up controls for primary arthroplasty patients |
| Evaluation of a smartphone-based assessment system in subjects with chronic ankle instability | 2017 | app for the assessment of postural control ability for patients with chronic ankle instability. |
| Smartphone assessment of knee flexion compared to radiographic standards | 2017 | app to determine the knee range of motion |
| The "Strengthen your ankle" program to prevent recurrent injuries: A randomized controlled trial aimed at long-term effectiveness | 2017 | evaluation whether the implementation method of a proven effective neuromuscular training program delivered by a mobile application or a written instruction booklet, resulted in differences in injury incidence rates or functional ankle disability/pain |
| Course of pelvic lift during total hip arthroplasty | 2017 | determination of the pelvic position during total hip athroplasty by using an app |
| Postoperative monitoring with a mobile application after ambulatory lumbar discectomy: an effective tool for spine surgeons | 2016 | mobile app for postoperative monitoring after outpatient lumbar discectomy. |
| USE OF SMARTPHONE IN ACETABULAR COMPONENT ANGLE MEASUREMENT DURING TOTAL HIP ARTHROPLASTY | 2016 | using a smartphone to measure the angle of acetabular component in total hip arthroplasty |
| Reliability and Criterion Validity of the Smartphone Inclinometer Application to Quantify Cervical Spine Mobility | 2017 | using a smartphone to measure cervical range of motion |
| Use of digital images to aid in the decision-making for acute upper extremity trauma referral | 2016 | use of digital smartphone images in the decision-making for acute upper extremity trauma referrals |
| Measurement properties of the smartphone-based B-B Score in current shoulder pathologies | 2015 | shoulder function B-B Score measured with a smartphone |
| The smartphone app 'Rotator Cuff Injury/Strain' by Medical iRehab | 2016 | explaining the iRehab app for rotator cuff injuries |
| Comparison between Oxford Cobbmeter and digital Cobbmeter for measurement of Cobb angle in adolescent idiopathic scoliosis | 2016 | comparison of the Oxford cobbmeter with a digital cobbmeter to measure the cobb angle in scoliosis |
| Doctors and the Etiquette of Mobile Device Use in Trauma and Orthopedics | 2015 | investigation on the influence of mobile device use on patient and staff opinions in the trauma and orthopedics department at a teaching hospital in Wales |
| Mobile-Web app to self-manage low back pain: randomized controlled trial | 2015 | app for treatment of unspecific low back pain |
| WhatsApp Messenger is useful and reproducible in the assessment of tibial plateau fractures: inter- and intra-observer agreement study | 2015 | evaluation of the inter- and intra-observer agreement in the initial diagnosis and classification by means of plain radiographs and CT scans of tibial plateau fractures photographed and sent via WhatsApp Messenger |
| Smartphone versus knee ligament arthrometer when size does not matter | 2014 | this study evaluated the performance of a portable device based on a downloadable electronic smartphone application to measure anterior tibial translation in ACL-deficient knees |
| Validity and reliability of the iPhone to measure rib hump in scoliosis | 2014 | using an app to measure the rib hump in scoliosis |
| Validity and intra-rater reliability of an android phone application to measure cervical range-of-motion | 2014 | using an app to measure cervical range of motion |
| A comparison of the reliability of the trochanteric prominence angle test and the alternative method in healthy subjects | 2014 | transcondylar angle test (TCAT) as an alternative to the trochanteric prominence angle test (TPAT) and using a smartphone as a reliable measurement tool for femoral neck anteversion (FNA) measurement. |
| Measurement of rotational deformity: using a smartphone application is more accurate than conventional methods | 2013 | assessment of the accuracy of three different methods measuring the angle between two fixed Kirschner wires for the potential purpose of determining correction during rotational osteotomy of long bones |
| The reliability, minimal detectable change and concurrent validity of a gravity-based bubble inclinometer and iphone application for measuring standing lumbar lordosis | 2014 | using an app to measure lumbar lordosis |
| Reliability and criterion validity of two applications of the iPhone™ to measure cervical range of motion in healthy participants | 2013 | using an app to measure cervical range of motion |
| Inter- and intra-observer reliability of a smartphone application for measuring hallux valgus angles | 2013 | using an app to determine different foot angles in patients with hallux valgus |
| Validation of a novel smartphone accelerometer-based knee goniometer | 2012 | smartphone based knee goniometer |
| Intraoperative measurement of femoral antetorsion using the anterior cortical angle method: a novel use for smartphones | 2013 | using a smartphone to measure the femoral antetorsion |
| Improving acetabular cup orientation in total hip arthroplasty by using smartphone technology | 2012 | using a smartphone to check the pelvic position in total hip arhroplasty |
| Reliability analysis of a smartphone-aided measurement method for the Cobb angle of scoliosis | 2012 | using a smartphone to measure cobb angle in scoliosis |
| Use of the iPhone for Cobb angle measurement in scoliosis | 2012 | using a smartphone to measure cobb angle in scoliosis |

Supplement 3: Overview of the 91 articles included and their brief content
